# Adverse childhood experiences and adolescent mental health: trajectories of internalizing, externalizing and prosocial behaviour in a longitudinal cohort study

**DOI:** 10.1017/S2045796026100808

**Published:** 2026-07-27

**Authors:** Iftikhar Ahmed Charan, Wang Chongjin, Shazia Soomro

**Affiliations:** 1 Public Administration, Shandong Technology and Business University, China; 2 Sociology, Anhui University, China

**Keywords:** adverse childhood experiences, externalizing behaviour, internalizing, longitudinal cohort study, Pakistan, prosocial behaviour

## Abstract

**Aims:**

Adverse childhood experiences (ACEs) shape children’s socioemotional development, yet longitudinal evidence from low- and middle-income countries remains limited. This study examined how ACEs relate to internalizing, externalizing and prosocial behaviours from childhood to adolescence in a rural Pakistani cohort.

**Methods:**

Data were drawn from the Bachpan Cohort, a longitudinal study including 1,791 observations across three waves: ages 4–5, 8–9 and 12–15 years. ACEs were assessed using an adapted ACE-International Questionnaire. Outcomes were measured using the Strengths and Difficulties Questionnaire. Network analysis, dose-response modelling and moderation analyses were employed.

**Results:**

Emotional neglect was the most prevalent ACE (83.7%). Parental psychiatric issues, parental trauma exposure and emotional neglect were the most central ACE stressors. A dose-response relationship emerged: compared to children with no ACEs, those with four or more ACEs showed higher internalizing (incidence ratio [IR] = 4.24) and externalizing problems (IR = 3.42) and lower prosocial behaviour (IR = 0.90). Girls showed greater internalizing vulnerability; boys showed more externalizing problems. ACE effects on internalizing problems strengthened with age.

**Conclusions:**

ACEs are strongly associated with internalizing, externalizing and prosocial outcomes from childhood to adolescence. Findings highlight the need for tiered, gender-sensitive and developmentally appropriate interventions in low-resource settings.

## Introduction

Adverse childhood experiences (ACEs) refer to severely stressful exposures or experiences that occur in childhood, such as abuse, neglect, violence between caregivers and peer or community violence (Chung *et al.*, 2023). Common examples of ACEs include child maltreatment abuse, conflict between parents, separation and psychological disorders among caregivers (Bevilacqua *et al.*, 2021). Childhood is a critical period when such traumatic events occur, affecting both a child’s present well-being and future health outcomes. A wider range of ACEs also covers economic hardship, physical and emotional mistreatment, sexual abuse, household conflict, substance problems and parental mental illness (Gautam *et al.*, 2024). The effects of early adversity appear shortly after exposure and persist over time. They first show up as developmental delays in childhood and eventually contribute to poor mental health and physical health in later adulthood (Cprek *et al.*, 2020; Chung *et al.*, 2023).

Exposure to severe stress early in life can change how the brain grows and works. These changes may have lasting effects on a child’s development and also affect body systems like immune function, stress responses and inflammation (Berens *et al.*, 2017). ACEs occur frequently across different populations. The dysregulations of the central nervous system, the endocrine system and the immune system that many victims of early trauma display are direct consequences of long-term exposure to toxic stress. These internal changes can stretch themselves as a number of psychological, emotional, behavioural and social problems that arise in children who begin growing up towards adulthood (Gautam *et al.*, 2024). Therefore, substantial efforts have been made to avert ACEs and diminish their adverse outcomes, particularly in child and adolescent samples (Herzog and Schmahl, 2018; Tonmyr *et al.*, 2020).

Recent research has repeatedly confirmed a robust link between primary ACEs experience and later development of depression and anxiety through childhood and adolescent years (Bellis *et al.*, 2016; Hughes *et al.*, 2016, 2017; Elmore and Crouch, 2020; Jones and Pierce, 2021; Jackson *et al.*, 2023). Furthermore, the relationship follows a clear dose-response pattern, where higher ACE exposure leads to greater risk (Hoffmann and Jones, 2022). Children who face multiple ACEs tend to develop poor emotion regulation skills, including heightened anxiety and depression, along with behavioural characteristics like anger and lack of empathy (Jones and Hoffmann, 2023). Consistent with these findings, research on at-risk adolescent populations has shown that those placed in residential care exhibit more emotional-behavioural problems and greater vulnerability in variables linked to emotional-behavioural problems, such as insecure-disorganized attachment and alexithymia (Muzi and Pace, 2023).

A growing body of research indicates that nearly 89% of young people experience at least one ACE during childhood. Experiencing multiple ACEs affects the amygdala, which raises the likelihood of developing several psychological disorders and physical health conditions (Holmes *et al.*, 2021; Loxton *et al.*, 2021; Malvaso *et al.*, 2022). Consequently, the neurological and emotional effects of prolonged ACE exposure in early life are strongly tied to greater risks of externalizing behaviours, internalizing problems and reduced prosocial behaviour among children and adolescents, with these effects continuing into later developmental periods (Herzog and Schmahl, 2018; Lee *et al.*, 2020; Hicks *et al.*, 2021).

Children whose mothers had more ACEs tend to show worse socioemotional functioning, including more internalizing and externalizing problems (Chung *et al.*, 2023). In the first three years of life, these socioemotional problems show up as higher levels of anxiety, aggression, hyperactivity and negative emotions (McDonnell and Valentino, 2016; Schickedanz *et al.*, 2018; Cooke *et al.*, 2019). Several factors make addressing ACEs in low- and middle-income countries critically important: childhood adversity occurs more frequently in these settings, resources for intervention are limited and most of the global population lives in low- and middle-income countries. Understanding how ACEs influence development across different cultural settings can assist in identifying vulnerable populations who require targeted interventions (McDonnell and Valentino, 2016; Letourneau *et al.*, 2019; Treat *et al.*, 2020).

Several studies of evidence have shown that the adverse effects of ACEs are observable during childhood and adolescence (Scully *et al.*, 2020). Like studies of adults, ACE exposure has been associated with internalizing behaviour problems and adolescent outcomes such as suicide (Bevilacqua *et al.*, 2021). According to more recent studies, anxiety and depression are categorized as disorders. In fact, these are disorders that involve the types of symptoms regarding internal emotional distress (Wergeland *et al.*, 2021; Mouro Ferraz Lima *et al.*, 2023). Common internalizing disorders include anxiety disorders and mood disorders. A large body of research has confirmed that exposure to ACEs increased the likelihood of developing internalizing disorders (Al-Rousan *et al.*, 2025). Extending this evidence, Pace *et al.* (2022) found that among late-adopted adolescents, maltreatment and its combination with multiple placements changes were strong predictors of emotional and behavioural problems, including social, thought and identity difficulties (Pace *et al.*, 2022).

Across different countries, research has found that the proportion of children and adults in general populations who have encountered at least one ACE ranges from 23 to 77% (Bellis *et al.*, 2019; Crouch *et al.*, 2019; Giano *et al.*, 2020; Subramaniam *et al.*, 2020; Turney, 2020; Kappel *et al.*, 2021; Zhou *et al.*, 2025). Children who experience adversities such as neglect, abuse and household dysfunction face a higher risk of developing children’s mental health and behavioural problems during both childhood and later adult years (Li *et al.*, 2020; Oei *et al.*, 2021). A growing number of studies have reported a dose-response relationship, meaning that a high number of ACEs is associated with greater risks for health and developmental problems (Webster, 2022). Findings from the UK Millennium cohort study showed that youth who experienced multiple ACEs had considerably worse mental health outcomes compared to those who reported only a single ACE (Bevilacqua *et al.*, 2021). Another study also documented a dose-response pattern, where cumulative childhood adversities were linked to more emotional and behavioural difficulties, poorer sleep quality and lower academic achievement (Qu *et al.*, 2024).

In the classifications of child behavioural problems, externalizing behaviours refer to actions such as aggression, conduct issues and challenges in social interaction (Shaheen *et al.*, 2023). In contrast, internalizing problems involve self-directed difficulties, including anxiety, depression and social withdrawal, which tend to be more emotionally painful and inwardly focused. Although considerable research has examined how ACEs relate to internalizing, externalizing and general emotional-behavioural problems across different age groups, much less attention has been given to whether ACEs affect adaptive or positive behaviours. Specifically, the possible impact of ACEs on the development of prosocial behaviour such as helping others, sharing, cooperating and showing kindness to peers, remains understudied (Bevilacqua *et al.*, 2021). Evidence suggests that prosocial behaviour typically increases until around age 14, yet greater exposure to adversity is linked to fewer prosocial actions over the course of development (Bevilacqua *et al.*, 2021).

Driven by the gaps in prior research, this study aims to investigate the longitudinal associations between ACEs association with internalizing, externalizing, emotional-behavioural problems and prosocial outcomes from childhood through adolescence. While extensive research has documented the harmful effects of ACEs on mental health, relatively few studies have examined how ACEs influence adaptive behaviours such as prosocial actions, including helping, sharing and cooperating with peers. Additionally, most existing research has relied on cross-sectional designs or retrospective reports of childhood adversity, which are known to introduce recall bias and may overestimate associations between ACEs and later outcomes.

To address these gaps, the present study extends previous research by: (a) identifying the most central ACE stressors using network analysis, (b) examining dose-response effects across multiple developmental stages and (c) testing gender and age moderation in a general population sample. Data are drawn from a prospective longitudinal cohort with multiple time points from childhood through adolescence. Behavioural outcomes were assessed using the Strengths and Difficulties Questionnaire (SDQ; Goodman, 1997), and ACEs were measured using the ACE-international Questionnaire (ACE-IQ), which has been validated across various cultural contexts with recent psychometric studies confirming its factor structure across gender and age groups (ACE-IQ; WHO, 2018; Muzi *et al.*, 2025). This study also examines whether associations differ by gender. Overall, this study offers a novel contribution to the existing literature by providing new insights into the longitudinal cohort mechanisms that explain how ACEs shape the development of internalizing, externalizing behaviours and prosocial behaviour in children and adolescents over time.

Specifically, we hypothesized that (H1) a higher prevalence of ACEs will be positively associated with greater internalizing and externalizing behaviours, emotional-behavioural problems and traumatic stress symptoms, but negatively associated with prosocial behaviours, reflecting a dose-response pattern. (H2) Specific ACE types, particularly parental psychiatric issues and emotional neglect, will show stronger associations with adverse outcomes compared to other adversity, (H3) Gender differences will be observed, with stronger associations between ACEs and internalizing problems among girls, and stronger associations between ACEs and externalizing problems among boys and (H4) Age will moderate the relationship between ACEs and outcomes, with effects strengthening from childhood to adolescence.

## Method

### Study setting

We used data from the Bachpan Cohort, which is a long-term study that follows children from birth. The cohort is based in Kallar Syedan, a rural area within Rawalpindi District, Punjab Province, Pakistan (Turner *et al.*, 2016; Maselko *et al.*, 2020; Chung *et al.*, 2023). The original cohort was designed to examine how perinatal depression and maternal mental health affect early child development. Information was collected from mothers as primary caregivers during pregnancy and at several follow-up time points after childbirth. For the present analysis, behavioural outcomes were assessed at three developmental stages: early childhood at ages 4–5 years (wave 3), middle childhood at ages 8–9 years (wave 5) and adolescence at ages 12–15 years (wave 8). The analytic dataset included 1,791 observations across these three waves: wave 3, *n* = 487; wave 5, *n* = 563; and wave 8, *n* = 741. Because the data were longitudinal and unbalanced, participants did not necessarily contribute data at every wave. Generalized estimating equation (GEE) models were therefore appropriate because they account for repeated observations within individuals over time.

## Dependent variables and outcome variables

### Measure of externalizing, internalizing and prosocial behaviour

The outcome variables in this study were externalizing, internalizing and prosocial behaviours, all measured using the SDQ (Goodman, 1997). The SDQ is a dependable instrument for evaluating the emotional, behavioural and social well-being of children and adolescents across five areas: hyperactivity, emotional difficulties, conduct problems, peer issues and prosocial behaviour (Goodman, 1997; Goodman *et al.*, 2000). An Urdu version of the SDQ has been developed and validated in Pakistan, showing evidence of both construct and discriminant validity. The measure is commonly used in low- and middle-income countries and has been shown to give consistent and accurate results across diverse cultural settings (Chung *et al.*, 2023).

In the Bachpan Cohort, externalizing behaviours were assessed using the SDQ. The hyperactivity subscale consisted of five items: restlessness, constant fidgeting, easy distractibility, thinking before acting and sustaining good attention. The conduct problems subscale also had five items: irritability, following instructions, fighting or bullying, arguing with adults and acting spitefully toward others. For externalizing behaviours, we used the emotional difficulties subscale (items included headaches, worrying, feeling sad, nervousness and fear) together with the peer problems subscale (items included playing by oneself, acceptance by other children, being bullied, adult relationships and having a close friend). In addition, prosocial behaviours (for example, willingness to share with peers, helping those who are injured, showing kindness to younger children and tendency to volunteer assistance) were assessed using the prosocial behaviour scale.

We recorded SDQ responses on a 3-point Likert scale: ‘Not true’, ‘Somewhat true’ and ‘Certainly true’. We created a total difficulty score by adding the first four domains (emotional problems, conduct problems, hyperactivity and peer problems). A higher overall score indicated more significant behavioural problems. The SDQ demonstrated good internal reliability, with Cronbach’s alpha values falling between 0.7 and 0.9 (Goodman *et al.*, 2000). Children with elevated SDQ scores exhibited a greater level of both externalizing and internalizing problems. Those with higher prosocial scores showed more positive social behaviours. Lower scores reflected better emotional and behavioural health, but also less prosocial behaviour in this group. We collected these measures from wave 3 through wave 8 (when children were 4 and 15 years old) and treated them as continuous variables in our analysis.

## Independent variables

### Measure of ACEs

Our main variables of interest were ACEs. To assess ACEs, we used an adapted form of the ACE-IQ, a retrospective self-report instrument that has exposed validity in low-resource settings. This adapted ACE-IQ originates from the original ACE measure developed in the United States (Felitti *et al.*, 1998) and was broadened to include items about peer violence, exposure to community violence and witnessing violence in the neighbourhood. Questions related to sexual abuse were excluded due to concerns about participant safety and anticipated underreporting. The ACE-QI has demonstrated satisfactory internal reliability and factorial validity in community samples (ACE-IQ; WHO, 2018; Muzi *et al.*, 2025). Several types of maltreatment fall under childhood adversity, such as psychological and physical abuse, together with problems in household functioning. Dysfunction within the home includes experiencing domestic violence, the death or separation of a parent, economic strain, disorders related to substance use, mental illness in caregivers and relatives having contact with the criminal legal system. These ACE checklists help researchers understand how early hardships affect children’s mental health and behaviour over time, including internalizing problems, externalizing problems and prosocial behaviour (Felitti *et al.*, 1998; Bellis *et al.*, 2016).

In line with a large body of work describing normative childhood stressors, the present analysis included adversities that have been widely studied in early childhood (Bethell *et al.*, 2017; O’Connor *et al.*, 2020; Bevilacqua *et al.*, 2021). Such adversities were also recorded as part of the Bachpan cohort, which started data collection in wave 3 at ages 4–5 years. All adversity categories explored in this study are listed in Table 1. We evaluated ACEs using a checklist within the Bachpan Cohort dataset that contained the following items. ACE data were at wave 3 (4–5 years) and again at wave 5 (8–9 years):

- Emotional neglect: Participants were asked about experiences of emotional neglect by their parents during childhood using a 5-point Likert scale ranging from ‘never or seldom’ to ‘often’. We split the response into two categories. Answers like ‘all the time’, ‘more than half the time’, ‘about half the time’, ‘less than half the time’ were marked as ‘Yes’. The option ‘never or rarely’ was marked as ‘No’.
- Physical neglect: Physical neglect was evaluated using four questions administered to both behaviour management, the level of emotional distress when handling the child’s behaviour, the influence of aggressive parenting behaviours on discipline practices, and the degree of feeling overwhelmed in managing the child’s behaviour. Responses were collected on 5-point Likert scale from ‘Never’ (1) to (5) ‘Many times each day’. A composite score was created and then dichotomized, with ‘Never’ classified as ‘No’ and all other responses (‘Many times per day’, ‘Once a week or less’, ‘One or two times per day’ and ‘A few times per week’) classified as ‘Yes’.
- Parental conflict: Parental conflict was measured using four questions posed to both mothers and fathers. These questions assessed how often families experience significant conflict due to traumatic events, how frequently the child’s behaviour leads to parental feelings of being overwhelmed or stressed, the extent to which family conflicts or crises cause distress and the level of anger or frustration resulting from traumatic family experience. Response was gathered using a 5-point Likert scale and collapsed into one binary variable. Answers like ‘Always’, ‘Often’, ‘Sometimes’ and ‘Rarely’ were counted as ‘Yes’, while ‘Never’ was counted as ‘No’.
- Parental divorce or segregation: To assess parental divorce or separation, parents were asked whether they had experienced separation due to relationship or marital problems. This information was recorded as a binary variable, where children who did not live with both biological parents were coded as ‘Yes’ and those living with both parents were coded as ‘No’.
- Parental mental illness: Parental mental health was assessed using the K6 depression scale. Parents were asked how often during the last 4 weeks they felt nervous, hopeless, restless or worthless, or unable to cheer up. Each response was recorded in a binary format, where ‘0’ meant that there was no probable serious mental illness and ‘1’ stood for the presence of serious mental illness.
- Family psychological distress: Family psychological distress was measured with the following question: ‘Have you experienced psychological trauma or significant emotional family difficulties resulting from family or other traumatic events?’ Responses were recorded as binary, with ‘Yes’ indicating that children had been exposed to emotional and psychological stress and ‘No’ indicating no such exposure.
- Emotional and psychological burden: Emotional and psychological burden was measured by asking participants: ‘In the past year, have you experienced any severe emotional or psychological concern, such as sadness, anxiety or overwhelming stress, due to parental or family issues?’ A response of ‘Yes’ confirmed the presence of such burden, while ‘No’ indicated its absence.
- Childhood emotional neglect: Childhood emotional neglect was evaluated using a single question: ‘in the past year, has your child experienced emotional neglect, meaning a lack of care, support or affection from you or other caregivers?’ Responses were coded as ‘0’ for no and ‘1’ for yes.
- Financial strain on families: Family financial strain was assessed using a six-item hardship scale completed by the primary caregiver. Responses were recorded in a yes/no format, with ‘1’ indicating the presence of financial pressures and ‘0’ indicating their absence.

**Table 1.** Study participant descriptive statistics Table 1 long description.

Variables	Wave 3 (4–5 yrs) *n* = 487	Wave 5 (8–9 yrs) *n* = 563	Wave 8 (12–15 yrs) *n* = 741	Pooled (4–15 yrs) *N* = 1,791
Outcome variables				
Externalizing behaviour	5.4	4.7	5.9	5.3
Internalizing behaviour	2.9	3.3	5.1	3.8
Prosocial behaviour	7.8	9.1	7.5	8.1
ACE stressors (%)				
Emotional neglect	87.1	85.6	80.9	83.7
Physical neglect	7.4	12.1	11.3	10.2
Parental conflict	6.1	7.4	7.3	6.9
Parental divorce/segregation	2.6	4.3	5.1	4.0
Parental mental illness	18.7	19.9	15.3	17.9
Family psychological distress	2.7	3.4	2.2	2.7
Parental psychological burden	3.2	3.5	4.9	3.8
Death of family member	3.9	6.2	7.5	5.9
Financial strain on families	1.9	2.7	3.8	2.8
Child cognitive-behavioural development	92.4	91.3	90.7	91.4
Psychosocial factor and trauma	13.4	14.1	15.2	14.3
Anxiety and behavioural issues	94.2	92.8	91.1	92.7
Demographic variables				
Age (yrs)	4.3	8.7	14.3	9.5
Gender – male (%)	51.1	51.5	51.8	51.4
Gender – female (%)	48.9	48.5	48.2	48.6
Household income (%)				
Limited income	17.3	10.4	6.8	11.4
Below-average income	45.8	28.9	21.3	31.9
Above-average income	31.4	49.1	53.7	44.2
Wealthier segment	5.5	11.6	18.2	12.5
Parent’s education status (%)				
No formal schooling	9.8	10.9	11.8	10.9
Primary school completion	27.4	29.6	32.1	29.8
Secondary school completion	61.2	58.4	53.8	57.7
Higher education	1.6	1.1	2.3	1.6
Parent’s employment status (%)				
Employed	66.3	77.1	88.2	77.4
Unemployed	2.4	3.1	2.7	2.7
Unskilled work	31.3	19.8	9.1	19.9

*Note*: Values represent means for continuous variables and percentages for categorical variables across three waves. Total pooled *N* = 1,791 (wave 3: *n* = 487; wave 5: *n* = 563; wave 8: *n* = 741).

ACEs scores were computed by summing the binary values assigned to each adversity (Webster, 2022). A cumulative ACE score was then derived by adding all binary indicators, yielding a possible total between 0 and 11 (Webster, 2022; Chung *et al.*, 2023). Using these cumulative scores, participants were divided into six groups: no ACEs, 1 ACE, 2 ACEs, 3 ACEs, 4 ACEs and 5 or more ACEs.

## Other variables

To reduce the risk of bias from outside factors, we included several demographic variables as covariates in our Poisson GEE models. These variables were chosen based on previous research showing they can influence both ACE exposure and child behavioural outcomes. First, we looked at the child’s age. We split age into three developmental stages: early childhood (4–5 years), middle childhood (8–9 years) and adolescence (12–15 years). This helped us see how ACE effects might change as children grow older. Second, we considered the child’s sex, which we recorded as male or female. Third, we looked at household income. Families reported their income in four categories: lowest, medium, medium-high and highest. Fourth, we accounted for the parents’ education level. This was grouped into four categories: no formal schooling, primary school completed, secondary school completed and higher education. Fifth, we included parents’ employment status, which told us whether the parents were working or not. Finally, we also considered the share of participants in each of these categories. This helped us better capture the range of differences present in our study sample.

## Statistical analysis

All data analyses were done using R (version 4.4.2). Descriptive statistics, which were initially used to compute frequencies, percentages and means for all relevant variables. For continuous measures such as age, externalizing scores, internalizing scores, and prosocial scores, means and standard deviations were calculated. For categorical measures, including all ACE indicators, gender, household income, parental education and parental employment, frequencies and percentages were computed. Table 1 presents the main descriptive findings. To examine how ACEs relate to internalizing problems, externalizing problems, stress, emotional and behavioural problems and prosocial behaviours over time, we fitted GEE models with a Poisson distribution. A key feature of the Poisson distribution is that its mean equals its variance. If the variance is greater than the mean, the data show overdispersion. We checked for this in our data and found that the mean and variance were close to equal, meaning overdispersion was not a concern. Given this, the Poisson model was a suitable choice for our analysis (Gardner *et al.*, 1995).

Latent class analysis was also applied to identify distinct patterns of ACE exposure. Following the guidelines of Nylund *et al.* (2007), model selection was guided by log-likelihood values, the Bayesian Information Criterion (BIC) and the Akaike Information Criterion (AIC) (Nylund *et al.*, 2007). Models with lower BIC, AIC and likelihood ratios were considered better fitting (Tein *et al.*, 2013; McLachlan *et al.*, 2019). Constructed on these criteria, five-class solution was identified. However, due to low entropy values indicating poor class separation, the LCA results were not used in the final analysis. ACEs’ interaction effects with gender and age group were also tested. These methods allowed us to investigate the associations between ACEs and four behavioural categories, externalizing, internalizing, conduct problems and prosocial behaviour across development from childhood to adolescence. We included these interaction terms to see if boys and girls had different effects from ACEs behaviour, as well as if the outcome varied by age group. It provided a better idea of how these connections change through time.

We tested several correlation structures for the GEE models, such as independent, exchangeable, autoregressive and unstructured. To choose the best fitting correlation structure, we applied the quadratic inference criterion (QIC) (Ballinger, 2004; Odueyungbo *et al*., 2008). A lower QIC value reflects a better fit for the model. The exchangeable correlation structure yielded the lowest QIC value and was consequently selected for all final models. Network analysis was conducted to determine which ACE stressors were most central in influencing behavioural outcomes. Several centrality metrics were calculated for each ACE stressor, including node strength, degree centrality, betweenness centrality, closeness centrality and expected influence. Based on composite scores across all metrics, an overall risk ranking was assigned to each ACE stressor. The results of these network analyses are shown in Table 2. Finally, the associations between ACE exposure and behavioural outcomes (internalizing, externalizing, emotional and behaviour problems and prosocial behaviours) were expressed as incidence ratios (IRs) with 95% confidence intervals (CIs). Statistical significance was defined as *p* < 0.05. Simple imputation techniques were applied to manage missing data.

## Ethical approval

The Bachpan Cohort study received ethical approval from the institutional review board at Human Development Research Foundation (IRB/1017), Duke University and the University of North Carolina at Chapel Hill (#20-1433). All procedures followed the ethical standards of the Declaration of Helsinki. All participants gave written informed consent before taking part. For those who could not read or write, a witness helped with the consent process.

## Results

### Descriptive statistics

Descriptive statistics for all variables included in the current study (*N* = 1,791, waves 3–8) can be found in Table 1. The results indicate that externalizing behaviours remain stable throughout development, with mean scores between 4.5 and 5.9. Internalizing behaviours increased from a mean score of 2.9, indicating low rates in the early years (ages 4–5 years), to an average of 5.1 by adolescence (ages 12–15 years), with a total overall mean of 3.8 for all waves together. Prosocial behaviours peak during middle childhood (ages 8–9 years) with a mean of 9.1 and subsequently decline to 7.5 by adolescence. Looking at ACEs, emotional neglect was very common across all age groups, with 87.1% of children aged 4–5 years reporting it, and the rate dropped modestly to 80.9% among adolescents. Parental mental illness problems impacted roughly 15–20% of the participants across the different waves.

Physical neglect showed an increasing trend from 7.4% in early childhood to 12.1% in middle childhood. Parental conflict and parental divorce or separation also increased with child age. Financial strain was most common among the youngest children at 1.9%, with a small rise seen in the older age brackets. Child cognitive-behavioural development difficulties and anxiety-related behavioural issues remain consistently high across all waves, exceeding 90% of the illustration. In relationships with respondents’ demographic characteristics, the proportion of males and females stayed fairly even across all age groups, with about 51% boys and 49% girls. Average household income rose as children got older, with the wealthiest category increasing from 5.5% during early childhood to 18.2% by adolescence, alongside rising levels of parental education and employment, as shown in Table 1.

### Network centrality of ACE stressors

Table 2 presents the network centrality metrics for each ACE stressor, identifying which adversities are most influential in shaping behavioural outcomes. The findings showed that parental psychiatric problems emerged as the most central ACE stressor. At 0.690, it had the strongest network and an overall risk rank of 1. Following this was parental trauma exposure, with a network strength of 0.560 at risk rank 2; emotional neglect was not far behind with a strength of 0.520, ranked 3 overall. Fourth, household legal challenges (strength = 0.460, rank = 4); fifth, parental divorce or separation (strength = 0.450, rank = 5); and sixth, financial stress (strength = 0.420, rank = 6). Conversely, the most peripheral ACEs were close relative loss (strength = 0.200, rank = 8), home drug exposure (strength = 0.280, rank = 7) and parental alcohol misuse (strength = 0.200, rank = 9). Parental alcohol use and loss of a close relative also both had non-zero betweenness centrality scores: 0.145 and 0.182, these values indicating that both play unique bridging roles within the ACE network.

**Table 2.** Network centrality metrics for ACE stressors (*N* = 1,791)

ACE stressor	Node strength (Σ|w|)	Degree centrality	Betweenness centrality	Closeness centrality	Expected influence (EI)	Overall risk rank
Emotional neglect	0.520	0.000	0.000	0.392	+0.380	**3**
Parental psychiatric issues	0.690	0.125	0.000	0.392	+0.510	**1**
Parental trauma exposure	0.560	0.250	0.000	0.392	+0.460	**2**
Parental divorce/segregation	0.450	0.375	0.000	0.392	+0.370	**5**
Financial strain	0.420	0.500	0.000	0.392	+0.300	**6**
Household legal challenges	0.460	0.625	0.000	0.392	+0.400	**4**
Loss of close relative	0.200	0.750	0.145	0.314	+0.200	**8**
Drug exposure in home	0.280	0.875	0.000	0.392	+0.200	**7**
Parental alcohol misuse	0.200	1.000	0.182	0.314	+0.120	**9**

Node Strength is defined as the sum of the absolute edge weights connected to a node, while Expected Influence (EI) is the sum of the signed edge weights. Degree, betweenness, and closeness are standard centrality indices used to characterize node importance in psychological networks (Robinaugh *et al*., 2016; Epskamp *et al*., 2018).

Bold values indicate the overall Risk Rank for each ACE stressor.

### Longitudinal association between ACE stressors and behavioural outcomes

The analyses were based on the network regression coefficients linking each ACE stressor to externalizing, internalizing and prosocial behaviours, which are shown in Table 3 externalizing behaviour variables, parental psychiatric conditions had the strongest positive correlation with child behavioural problems (*β* = 0.33, *p* < 0.01), followed by parental trauma exposure (*β* = 0.22, *p* < 0.05) and household legal difficulties (*β* = 0.17, *p* < 0.05). Significant positive associations were also observed for emotional neglect (*β* = 0.14, *p* < 0.05), parental divorce or segregation (*β* = 0.18, *p* < 0.05), financial strain (*β* = 0.16, *p* < 0.05), drug exposure in the home (*β* = 0.11, *p* < 0.01) and parental alcohol misuse (*β* = 0.13, *p* < 0.01).

**Table 3.** ACE associations with behavioural outcomes – network-augmented (*N* = 1,791) Table 3 long description.

ACE stressor	Externalizing *β* (SE)	Internalizing *β* (SE)	Prosocial *β* (SE)	Network strength	Network risk rank
Emotional neglect	0.14 (0.03)**	0.31 (0.04)***	−0.07 (0.02)**	**0.520**	**Moderate**
Parental psychiatric issues	0.33 (0.02)***	0.27 (0.03)***	−0.09 (0.03)***	**0.690**	**Highest**
Parental trauma exposure	0.22 (0.04)**	0.29 (0.04)***	−0.05 (0.01)*	**0.560**	**High**
Parental divorce/segregation	0.18 (0.01)**	0.23 (0.02)***	−0.04 (0.02)*	**0.450**	**Moderate**
Financial strain	0.16 (0.01)**	0.20 (0.04)**	−0.06 (0.03)***	**0.420**	**Moderate**
Household legal challenges	0.17 (0.03)**	0.26 (0.01)**	−0.03 (0.02)	**0.460**	**Moderate**
Loss of close relative	0.07 (0.04)	0.11 (0.03)*	0.02 (0.03)	**0.200**	**Low**
Drug exposure in home	0.11 (0.01)***	0.13 (0.03)	−0.04 (0.02)**	**0.280**	**Low**
Parental alcohol misuse	0.13 (0.03)***	−0.04 (0.03)	0.03 (0.04)*	**0.200**	**Low**

* *p* < 0.10, **p < 0.05, ***p < 0.01. Network risk rank derived from composite centrality index. ACE outcomes measured ages 12–15; gender reference = male.

Bold values indicate the Network Strength and Network Risk Rank values.

For internalizing behaviours, emotional neglect (*β* = 0.31, *p* < 0.01), parental trauma exposure (*β* = 0.29, *p* < 0.01) and parental psychiatric issues (*β* = 0.27, *p* < 0.01) showed the strongest positive associations. Household legal challenges (*β* = 0.26, *p* < 0.05), parental divorce segregation (*β* = 0.23, *p* < 0.01) and financial strain (*β* = 0.20, *p* < 0.05) also demonstrated significant effects, while loss of a close relative was marginally significant (*β* = 0.11, *p* < 0.10). Looking at prosocial behaviour, most types of ACEs were linked to lower scores. Strongest negative links were for parental mental health problems (*β* = −0.09, *p* < 0.01), emotional neglect (*β* = −0.07, *p* < 0.05) and money problems in the family (*β* = −0.06, *p* < 0.01). Drug use at home also had a clear negative link (*β* = −0.04, *p* < 0.05). Interestingly, parents drinking alcohol was weakly but positively linked to prosocial behaviour (*β* = 0.03, *p* < 0.10). This odd finding needs more study.

### Longitudinal dose-response relationships between cumulative ACEs and behavioural outcomes

**Figure 1.**
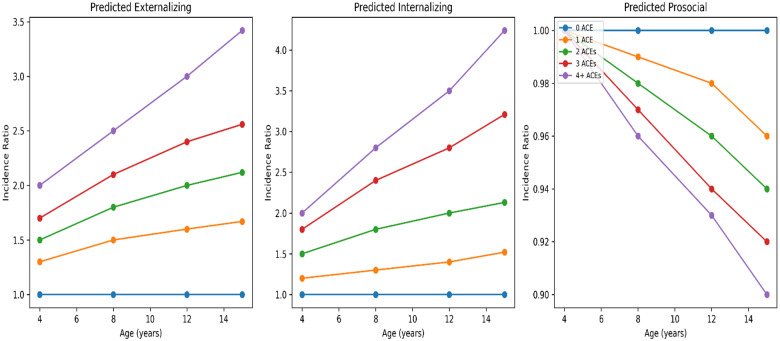
Predicted behavioural outcomes by ACE exposure level from ages 4 to 15 years. The three panels show externalizing behaviours, internalizing behaviours and prosocial behaviours across five ACE categories (0, 1, 2, 3 and 4+ stressors). Higher ACE exposure was associated with increased externalizing and internalizing problems and decreased prosocial behaviours over time. The dose-response relationship was strongest for internalizing outcomes. Figure 1 long description.

Table 4 and Fig. 1 show how the number of ACEs relates to behaviour problems from childhood through adolescence. Across all three outcomes, the pattern was clear: more ACEs led to worse outcomes. Take externalizing problems. Young people with just one ACE had a higher risk than those with no ACEs (adjIR = 1.67; 95% CI: 1.43–1.95; *p* < 0.01) when compared to peers without any ACE exposure. Participants with two ACEs demonstrated a 2.12 times greater risk (adjIR = 2.12; 95% CI: 1.80–2.49; *p* < 0.01), for three ACEs showed a 2.56 times greater risk (adjIR = 2.56; 95% CI: 2.06–3.19; *p* < 0.01), for four or more ACEs, the risk jumped to 3.42 times higher risk of externalizing difficulties (adjIR = 3.42; 95% CI: 2.36–4.91; *p* < 0.01).

**Table 4.** Longitudinal ACE dose–response – incidence ratios (*N* = 1,791) Table 4 long description.

	Externalizing behaviours		Internalizing behaviours		Prosocial behaviour	
Variables	Crude IR (95% CI)	Adj IR (95% CI)	Crude IR (95% CI)	Adj IR (95% CI)	Crude IR (95% CI)	Adj IR (95% CI)
ACE stressor accumulation						
No early stressors	1	1	1	1	1	1
Single stressor	1.68 (1.44–1.96)	1.67 (1.43–1.95)	1.51 (1.28–1.79)	1.52 (1.30–1.78)	0.96 (0.92–0.99)	0.96 (0.93–0.99)
Dual stressors	2.14 (1.82–2.52)	2.12 (1.80–2.49)	2.18 (1.74–2.73)	2.13 (1.72–2.64)	0.93 (0.88–0.96)	0.94 (0.91–0.97)
Triple stressors	2.59 (2.07–3.24)	2.56 (2.06–3.19)	3.27 (2.43–4.40)	3.21 (2.38–4.32)	0.91 (0.83–0.99)	0.92 (0.89–0.96)
4+ stressors	3.47 (2.41–4.99)	3.42 (2.36–4.91)	4.31 (2.64–6.82)	4.24 (2.58–6.71)	0.89 (0.80–1.06)	0.90 (0.87–0.94)
Gender moderation (reference: male)						
Single × Female	0.98 (0.94–1.02)	0.97 (0.93–1.01)	1.26 (1.19–1.34)	1.27 (1.21–1.35)	1.03 (1.01–1.05)	1.02 (1.01–1.04)
Dual × Female	0.96 (0.89–1.03)	0.96 (0.90–1.03)	1.31 (1.24–1.39)	1.32 (1.25–1.40)	1.02 (0.99–1.05)	1.01 (0.99–1.04)
Triple × Female	0.94 (0.85–1.04)	0.95 (0.86–1.04)	1.47 (1.39–1.56)	1.46 (1.37–1.55)	1.03 (0.99–1.07)	1.02 (0.99–1.06)
4+ × Female	0.79 (0.68–0.92)	0.80 (0.69–0.92)	1.83 (1.74–1.92)	1.84 (1.78–1.94)	1.04 (1.00–1.09)	1.04 (1.00–1.08)
Age moderation						
Single × Age	0.83 (0.78–0.88)	0.82 (0.77–0.87)	1.51 (1.45–1.57)	1.50 (1.44–1.56)	1.01 (0.99–1.03)	1.01 (0.99–1.03)
Dual × Age	0.76 (0.71–0.82)	0.77 (0.72–0.83)	1.71 (1.63–1.79)	1.70 (1.62–1.78)	1.02 (1.00–1.04)	1.02 (1.00–1.04)
Triple × Age	0.74 (0.67–0.82)	0.73 (0.66–0.81)	1.79 (1.69–1.90)	1.77 (1.68–1.88)	1.01 (0.97–1.05)	1.02 (0.98–1.05)
4+ × Age	0.66 (0.56–0.78)	0.67 (0.57–0.78)	1.89 (1.82–2.31)	1.87 (1.80–2.29)	0.97 (0.91–1.05)	0.98 (0.92–1.04)

*Note*: IR, incidence ratio. Adj IR adjusted for gender, ethnicity and parental education. Age categories: early childhood (4–5 yrs), middle childhood (8–9 yrs), adolescence (12–15 yrs). Pooled *N* = 1,791.

For internalizing problems, young people with a single ACE showed a significantly higher probability of internalizing problems (adjIR = 1.52; 95% CI: 1.30–1.78; *p* < 0.01), which rose to a 2.13 times greater risk for two ACEs (adjIR = 2.13; 95% CI: 1.72–2.64; *p* < 0.01), a 3.21 times greater risk for three ACEs (adjIR = 3.21; 95% CI: 2.38–4.32; *p* < 0.01) and a 4.24 times greater risk for four or more ACEs (adjIR = 4.24; 95% CI: 2.58–6.71; *p* < 0.01). The steep increase from three to four or more ACEs (from 3.21 to 4.24) suggests a potential threshold effect for internalizing problems. For prosocial behaviour, the opposite happened. One ACE lowered prosocial behaviour (adjIR = 0.96; 95% CI: 0.93–0.99; *p* < 0.05). Two ACEs lowered it more (adjIR = 0.94; 95% CI: 0.91–0.97; *p* < 0.01). Three ACEs lowered its future (adjIR = 0.92; 95% CI: 0.89–0.96; *p* < 0.01). Four or more ACEs lowered it the most (adjIR = 0.90; 95% CI: 0.87–0.94; *p* < 0.01).

Figure 1 shows how the number of ACEs relates to behavioural outcomes from age 4 to 15. The three graphs exhibition predicted scores for externalizing behaviours, internalizing behaviours and prosocial behaviours based on five ACE levels (0, 1, 2, 3 and 4 or more stressors). A clear pattern appears across all three outcomes. Children with more ACEs had worse results. Those with four or more ACEs consistently showed the highest levels of externalizing and internalizing problems. Children with no ACEs had the lowest levels of behavioural difficulties. Looking at externalizing behaviours, problems like aggression and hyperactivity rose steadily as ACEs increased. The rise was steepest for children with four or more ACEs, especially during adolescence.

For externalizing behaviours, the patterns were even stronger. Emotional problems like anxiety, depression and social withdrawal increased sharply with more ACEs. This suggests that repeated childhood adversity hits emotional well-being the hardest as children grow older. Prosocial behaviours, the opposite happened. Children with no ACEs showed the highest levels of helping, sharing and cooperation. As ACEs increased, prosocial behaviour went down. Children with multiple stressors had the lowest scores over time. Overall, the figure shows that more ACEs lead to worse behavioural and emotional outcomes from childhood through adolescence. These patterns offer strong evidence that early life stress had lasting effects, especially on emotional and behavioural adjustment.

Table 4 also shows gender differences, with boys as the reference group. Girls and boys responded differently to ACEs. For internalizing behaviours, girls showed progressively higher vulnerability to internalizing problems as ACE exposure increased, with girls having one ACE showing 1.27 times higher internalizing problems than boys (adjIR = 1.27; 95% CI: 1.21–1.35; *p* < 0.01), increasing to 1.32 times for two ACEs (adjIR = 1.32; 95% CI: 1.25–1.40; *p* < 0.01), 1.46 times for three ACEs (adjIR = 1.46; 95% CI: 1.37–1.55; *p* < 0.01) and 1.84 times for four or more ACEs (adjIR = 1.84; 95% CI: 1.78–1.94; *p* < 0.01). For externalizing problems, the opposite was true. Girls with four or more ACEs had lower risk than boys (adjIR = 0.80; 95% CI: 0.69–0.92; *p* < 0.01). For one to three ACEs, there were no clear gender differences. For prosocial behaviour, girls scored slightly higher than boys, but the differences were small and not significant. The IRs ranging from 1.01 to 1.04 across ACE levels, and all *p*-values were above 0.05.

Age also changed how affected outcomes, as shown in Table 4. For internalizing problems, the harmful effects of ACEs strengthened progressively with age. Compared to early childhood (reference), older children with one ACE showed 1.50 times higher internalizing problems (adjIR = 1.50; 95% CI: 1.44–1.56; *p* < 0.01), increasing to 1.70 times for two ACEs (adjIR = 1.70; 95% CI: 1.62–1.78; *p* < 0.01), 1.77 times for three ACEs (adjIR = 1.77; 95% CI: 1.68–1.88; *p* < 0.01) and 1.87 times for four or more ACEs (adjIR = 1.87; 95% CI: 1.80–2.29; *p* < 0.01). For externalizing problems, age worked as a protective factor. Older children demonstrating had lower risks than younger children. For one ACE, the risk was 0.82 (adjIR = 0.82; 95% CI: 0.77–0.87; *p* < 0.01), for two ACEs was 0.77 (adjIR = 0.77; 95% CI: 0.72–0.83; *p* < 0.01), for three ACEs, it was 0.73 (adjIR = 0.73; 95% CI: 0.66–0.81; *p* < 0.01) and for four or more ACEs, it was 0.67 (adjIR = 0.67; 95% CI: 0.57–0.78; *p* < 0.01). For prosocial behaviour, age did not make a meaningful difference. All interaction terms were close to 1.00 and not significant.

Overall, our findings emphasize the profound influence of ACEs on the behavioural, emotional and prosocial development of young people. The most central ACE stressors were parental psychiatric issues, parental trauma exposure and emotional neglect. A clear dose-response relationship was observed across all outcomes, with the strongest effects for internalizing problems at four or more ACEs. Gender and age significantly moderated these relationships, with girls showing heightened vulnerability to internalizing problems and older children demonstrating stronger internalizing effects but weaker externalizing effects.

## Discussion

Adverse childhood experiences profoundly shape child and adolescent development, yet their long-term effect on behavioural and emotional outcomes in low- and middle-income countries remains unexplored. The current study provides longitudinal evidence from a rural Pakistani cohort demonstrating that ACEs are linked to increased internalizing and externalizing problems and decreased prosocial behaviour from childhood through adolescence. On average, children with four or more ACEs showed more than four times higher risk for internalizing problems and more than three times risk for externalizing problems compared to peers with no ACE exposure. Parental psychiatric issues, parental trauma exposure and emotional neglect emerged as the most central ACE stressors. Girls showed greater vulnerability to internalizing problems, while boys showed more externalizing problems. The effects of ACEs on internalizing problems also strengthened with age. Taken together, these findings establish a benchmark for future research on ACEs and behavioural outcomes in low-resource settings.

As our hypothesis, higher ACE scores were linked to more externalizing and internalizing problems compared to children with no ACE exposure. This matches earlier studies showing that more ACEs increase the risk for both externalizing issues (like aggression) and internalizing issues (like emotional problems) (Scully *et al.*, 2020; Bevilacqua *et al.*, 2021; Gautam *et al.*, 2024). Research on other at-risk youth has reported similar patterns. For example, Muzi and Pace (2023) found that adolescents living in residential care had higher rates of externalizing and internalizing problems, more attachment insecurity and greater alexithymia than both late-adopted and community youth. These adolescents also showed the poorest outcomes across all measure domains, which is consistent with our findings that children with higher ACEs exposure exhibited progressively worse behavioural outcomes (Muzi and Pace, 2023).

We also found that children and adolescences with higher number of ACEs had a fewer prosocial behaviour. One reason may be that early adversity shows young people negative ways of acting and damages their self-worth. This can make it harder for them to think clearly before acting and to control their impulses. Over time, these difficulties can lead them to engage in more dangerous or hurtful behaviours toward others. These findings align with previous work showing that difficult family circumstances, including poor parenting, parental mental health issues, family disruption and financial pressure, are associated with diminished prosocial behaviour (Bevilacqua *et al.*, 2021). More recent studies have also highlighted how family characteristics, including prosocial behaviours, influence the mental health of children and adolescents (Watrous *et al.*, 2024).

Additionally, we explored how gender affects the relationship between ACE scores and externalizing, internalizing and prosocial behaviours. The boys and girls responded differently. The boys were more on externalizing problems, and the girls were more on internalizing problems. These findings are consistent with the report of Muzi and Pace (2023). Girls in their study always had more serious internalizing problems, and boys had externalizing problems (Muzi and Pace, 2023).

This supports the idea that gender differences are real and consistent. A mix of biological factors and how children are socialized probably explains these patterns. Earlier research reported similar patterns (Kring and Gordon, 1998; Eschenbeck *et al.*, 2007; Chaplin and Aldao, 2013; Godinet *et al.*, 2014). As a result, externalizing behaviour frequently reflects an intense response tied to ACEs and is observed more commonly among boys, while emotional and internalizing difficulties are more common in girls (Leban, 2021). In line with previous research showing that girls tend to have greater empathy and prosocial behaviour, our study also found that girls scored slightly higher than boys on prosocial measures (Watrous *et al.*, 2024).

Our findings showed that parental psychiatric problems, parental trauma exposure and emotional neglect exerted the strongest influence on externalizing, internalizing and prosocial actions. Previous research has also noted connections between parental mental health issues and a later increase in child behavioural problems (Bevilacqua *et al.*, 2021), and studies focused on at-risk populations have similarly reported cumulative effects. Recent research reported that attachment insecurity and alexithymia difficulties in identifying and describing emotions cumulatively predicted 25–43% of internalizing and 19–43% of externalizing problems across all groups (Muzi and Pace, 2023). These problems appear through visible signs like aggression, behavioural problems and emotional struggles, all of which negatively affect the environment around children and adolescents.

On the other hand, emotional neglect emerged as the third most central ACE stressor, with high prevalence across all developmental stages. When caregiving provides inconsistent care, fails to offer emotional support or shows little affection, children may become confused and struggle with regulating their emotions. Prior research found that emotional and physical abuse emerged as the primary drivers of the association between ACEs and adolescents’ adjustment difficulties. Extensive research supports the detrimental consequences of harsh parenting, which involves both physical and verbal aggression toward adolescents (Ksinan Jiskrova *et al.*, 2026). Research has demonstrated that parenting behaviours contribute to internalizing problems in adolescents by reducing their sense of self-worth and self-esteem and by encouraging maladaptive thinking patterns (Cole *et al.*, 2016; Li *et al.*, 2023). Overall, our findings highlight how essential stable caregiving, emotional support and clear boundaries are for healthy child development, while also stressing the importance of interventions aimed at preventing ACEs and reducing their negative effects.

We further demonstrated that ACEs were more predictive of internalizing problems in later adolescence compared to childhood, whereas their association with externalizing problems diminished. This developmental trajectory implies that the effects of early adversity may become more distinct across adolescence as children face increased social, academic and emotional challenges. Specifically, children exposed to ACEs may enter adolescence with a diminished emotional buffer and heightened stress reactivity. The present study fills an important gap in the literature on ACEs by examining how ACEs relate to socioemotional development among children and adolescents in Pakistan, a population group that has so far been excluded from work on ACEs. The study also adds to existing research on ACEs and how they relate to anxiety, aggression towards adults and the ability of subjects to help others. Specifically, because it was conducted in a non-Western context, crucially, these were the same individuals followed up over multiple years, so there is longitudinal data to explore whether early trauma effects persist or grow as a function of late adolescence age.

This study contributes to the existing literature by examining how ACEs relate to behavioural problems and prosocial behaviours over a two year in a low- and middle-income country setting. The associations between parental psychiatric problems, emotional neglect and household dysfunction and these behavioural outcomes suggest parental involvement in this literature can be targeted with multisectoral strategies (clinical services, community programs and parenting support (home visits); a combination of cash transfers for families co-administered with education and livelihood training; social service programming; psychological intervention; and school-based programs). These programs should focus on building social skills, as well as strengthening family relationships and units, especially for children whose parents have mental health issues. However, some ACE indicators (e.g., sexual abuse) were not available in the Bachpan Cohort dataset, as it is a secondary data study. Consequently, this void may constrain the breadth of generalizability for such findings. Despite these limitations, we achieved strong results consistent with the literature.

## Implications

Several practical directions follow from these findings. First, given that parental psychiatric issues and trauma exposure were the most central adversities, identifying and supporting caregivers with mental health difficulties could improve child outcomes across multiple domains. Integrating mental health screening into routine paediatric care, home visiting programs and early childhood settings would help reach affected families. Second, the high prevalence and strong influence of emotional neglect require that professionals working with children receive training to recognize this often-overlooked adversity. Interventions promoting emotional attunement, responsive caregiving and positive parent-child interaction may be especially valuable. Third, the clear dose-response pattern supports a tiered intervention approach. Universal programs promoting positive parenting may suffice for families with no or minimal adversity. Families with two to three adversities may benefit from targeted, evidence-based parenting programs. Families with four or more adversities likely require intensive, multi-component support addressing caregiver mental health, basic needs, parenting skills and child social-emotional development simultaneously.

Fourth, gender differences in response to adversity indicate that interventions should be tailored to how boys’ and girls’ express distress. Girls may need support managing anxiety, low mood and ruminative coping. Boys may need support in regulating anger and finding non-aggressive ways to express distress. Fifth, the strengthening of internalizing problems with age suggests that adolescent mental health services require adequate resources, though intervening earlier may be more effective than waiting until problems become severe. Finally, the decline in prosocial behaviour with increasing adversity reinforces the importance of actively teaching positive social skills, not merely reducing problem behaviours. School-based programs that build empathy, cooperation and conflict resolution skills may be particularly important for children growing up in stressful environments.

## Strengths and limitations

This study has several strengths. The longitudinal design followed children across three developmental stages, allowing examination of how adversity effects change with age. Network analysis moved beyond cumulative risk models to identify which specific adversities carry the most weight. Including prosocial behaviour alongside internalizing and externalizing outcomes expanded the focus beyond psychopathology to include positive adaptation. The sample was drawn from a rural low- and middle-income country where longitudinal data on child development remain scarce.

Several limitations must be acknowledged. First, despite the longitudinal design, causal claims cannot be firmly established because unmeasured factors may influence both adversity exposure and child outcomes. Second, adversities were assessed through parent report, which may be affected by recall bias or underreporting. Third, child outcomes for younger participants were also parent-reported, raising the possibility of shared method variance, though adolescents provided self-reports. Fourth, the sample came from one rural region of Pakistan; findings may not extend to urban populations or other cultural settings.

Fifth, the Bachpan Cohort did not include certain ACE items (e.g., sexual abuse) due to cultural sensitivity concerns, meaning our adversity assessment does not capture the full range of potential childhood adversities (Chung *et al.*, 2023). Sixth, some participants were lost to follow-up, though analyses revealed no systematic differences between those who remained and those who dropped out. Seventh, we did not examine mediating pathways (e.g., emotion regulation or parenting practices) that may explain how adversities translate into behavioural outcomes.

## Conclusion

This study gives strong evidence from a rural Pakistani longitudinal cohort study. It shows that single and multiple ACEs are tied to behavioural problems, emotional issues and social difficulties in young people. Our findings also confirm that ACEs play a major role in these struggles in children and adolescents. The identification of parental psychiatric issues, parental trauma exposure and emotional neglect as the most central ACE stressors offers clear targets for intervention. The dose-response relationship supports tiered intervention approaches, with higher ACE exposure requiring more intensive services. Gender and age significantly moderate the effects of ACEs, highlighting the need for targeted, developmentally appropriate and gender-sensitive interventions.

The current findings advance the very limited understanding in this area and have important clinical and policy implications. Especially for mental health trajectories in later life, it is important to consider the timing and nature of early stressors. Next, we can tailor suitable interventions for individual families, such as parenting programs, financial assistance, social services, mental health treatment, school-based interventions and community resources. Provide mental health aids for caregivers; drive flexibility of support systems covering emotional neglect; and promote and implement universal, comprehensive health and economic supports focused on families and communities. Furthermore, we also observe evidence consistent with some of the recommendations by child advocate organizations. These recommendations will allow better protection of the rights and well-being of young people and establish a basis for positive developmental pathways.

## Data Availability

The authors are not permitted to share the data.

## Table 1 Long description

Participant characteristics are summarized across three study waves spanning early childhood to adolescence, plus a pooled total, using means for continuous measures and percentages for categorical measures. Internalizing behavior increases from 2.9 at ages 4 to 5 to 5.1 at ages 12 to 15, while externalizing behavior dips at ages 8 to 9 then rises to 5.9 in adolescence. Pro-social behavior peaks at ages 8 to 9 at 9.1 and is lower at ages 12 to 15 at 7.5. Among ACE stressors, emotional neglect is consistently high but declines over time, from 87.1 percent to 80.9 percent; physical neglect increases from 7.4 percent to 11.3 percent, and parental divorce or separation rises from 2.6 percent to 5.1 percent. Death of a family member increases from 3.9 percent to 7.5 percent, and financial strain rises from 1.9 percent to 3.8 percent. Demographics are stable by gender at about half male and half female, while mean age progresses from 4.3 to 14.3 years. Household income shifts toward higher categories over time, with above-average income increasing from 31.4 percent to 53.7 percent and the wealthier segment from 5.5 percent to 18.2 percent, alongside declines in limited and below-average income. Parent employment shows more employed and fewer unskilled work over time, rising from 66.3 percent employed at ages 4 to 5 to 88.2 percent at ages 12 to 15. Comparisons across waves should be interpreted with awareness that sample sizes differ by wave and the pooled column combines all ages.

Navigate back to Table 1.

## Table 3 Long description

Three line graphs are shown. The first graph, titled ′Predicted Externalizing,′ shows age in years on the x-axis from 4 to 15 and incidence rate on the y-axis from 1.0 to 3.5. Lines represent different ACE categories: 0, 1, 2, 3 and 4 plus stressors. The incidence rate increases with age, with the highest rate for 4 plus stressors. The second graph, ′Predicted Internalizing,′ has the same axes. The incidence rate also increases with age, with 4 plus stressors showing the highest rate. The third graph, ′Predicted Prosocial,′ shows a decrease in incidence rate with age, with 4 plus stressors having the lowest rate. A legend differentiates ACE categories using distinct lines.

Navigate back to Table 3.

## Table 4 Long description

Incidence ratios compare behavioral outcomes across levels of ACE stressor accumulation, with crude and adjusted estimates reported for externalizing, internalizing, and prosocial behavior. Relative to no early stressors, externalizing rises stepwise from 1.68 with a single stressor to 3.47 with four or more stressors (adjusted values are very similar). Internalizing shows a steeper dose response, increasing from 1.51 with a single stressor to 4.31 with four or more stressors, again with minimal change after adjustment. Prosocial behavior trends slightly downward as stressors increase, from the reference level at no stressors to about 0.96 with a single stressor and about 0.89 to 0.94 at higher stressor counts, with the four or more category showing the widest uncertainty. Gender moderation terms (female compared with male) suggest little difference for externalizing at lower stressor counts, but lower externalizing at four or more stressors, while internalizing is consistently higher for females and increases with stressor level. Age moderation terms indicate externalizing associations weaken with age, whereas internalizing associations strengthen with age; prosocial age interactions are near no change. Estimates are observational and include confidence intervals, so precision varies across rows, especially at the highest stressor level.

Navigate back to Table 4.

## Figure 1 Long description

Three line graphs are shown. The first graph, titled ′Predicted Externalizing,′ shows age in years on the x-axis from 4 to 15 and incidence rate on the y-axis from 1.0 to 3.5. Lines represent different ACE categories: 0, 1, 2, 3 and 4 plus stressors. The incidence rate increases with age, with the highest rate for 4 plus stressors. The second graph, ′Predicted Internalizing,′ has the same axes. The incidence rate also increases with age, with 4 plus stressors showing the highest rate. The third graph, ′Predicted Prosocial,′ shows a decrease in incidence rate with age, with 4 plus stressors having the lowest rate. A legend differentiates ACE categories using distinct lines.

Navigate back to Figure 1.
